# Gene Expression Alterations Associated with Oleuropein-Induced Antiproliferative Effects and S-Phase Cell Cycle Arrest in Triple-Negative Breast Cancer Cells

**DOI:** 10.3390/nu12123755

**Published:** 2020-12-07

**Authors:** Samia S. Messeha, Najla O. Zarmouh, Abrar Asiri, Karam F. A. Soliman

**Affiliations:** 1 Division of Pharmaceutical Sciences, College of Pharmacy & Pharmaceutical Sciences, Florida A&M University, Tallahassee, FL 32307, USA; samia.messeha@famu.edu (S.S.M.); abrar1.asiri@famu.edu (A.A.); 2 College of Medical Technology-Misrata, Libyan National Board for Technical & Vocational Education, Misrata LY72, Libya; najlazar@yahoo.com

**Keywords:** oleuropein, triple-negative breast cancer, MDA-MB-231, MDA-MB-468, apoptosis, cell cycle, gene expression

## Abstract

It is known that the Mediterranean diet is effective in reducing the risk of several chronic diseases, including cancer. A critical component of the Mediterranean diet is olive oil, and the relationship between olive oil consumption and the reduced risk of cancer has been established. Oleuropein (OL) is the most prominent polyphenol component of olive fruits and leaves. This compound has been shown to have potent properties in various types of cancers, including breast cancer. In the present study, the molecular mechanism of OL was examined in two racially different triple-negative breast cancer (TNBC) cell lines—African American (AA, MDA-MB-468) and Caucasian American (CA, MDA-MB-231). The data obtained showed that OL effectively inhibits cell growth in both cell lines, concomitant with S-phase cell cycle arrest-mediated apoptosis. The results also showed that OL-treated MDA-MB-468 cells were two-fold more sensitive to OL antiproliferative effect than MDA-MB-231 cells were. At lower concentrations, OL modified the expression of many apoptosis-involved genes. OL was more effective in MDA-MB-468, compared to MDA-MB-231 cells, in terms of the number and the fold-change of the altered genes. In MDA-MB-468 cells, OL induced a noticeable transcription activation in fourteen genes, including two members of the caspase family: caspase 1 (*CASP1*) and caspase 14 (*CASP14*); two members of the TNF receptor superfamily: Fas-associated via death domain (*FADD*) and TNF receptor superfamily 21 (*TNFRSF21*); six other proapoptotic genes: growth arrest and DNA damage-inducible 45 alpha (*GADD45A*), cytochrome c somatic (*CYCS*), BCL-2 interacting protein 2 (*BNIP2*), BCL-2 interacting protein 3 (*BNIP3*), BH3 interacting domain death agonist (*BID*), and B-cell lymphoma/leukemia 10 (*BCL10*); and the CASP8 and FADD-like apoptosis regulator (*CFLAR*) gene. Moreover, in MDA-MB-468 cells, OL induced a significant upregulation in two antiapoptotic genes: bifunctional apoptosis regulator (*BFAR*) and B-Raf proto-oncogene (*BRAF*) and a baculoviral inhibitor of apoptosis (IAP) repeat-containing 3 (*BIRC3*). On the contrary, in MDA-MB-231 cells, OL showed mixed impacts on gene expression. OL significantly upregulated the mRNA expression of four genes: *BIRC3*, receptor-interacting serine/threonine kinase 2 (*RIPK2*), TNF receptor superfamily 10A (*TNFRSF10A*), and caspase 4 (*CASP4*). Additionally, another four genes were repressed, including caspase 6 (*CASP6*), pyrin domain (PYD), and caspase recruitment domain (CARD)-containing (*PAYCARD*), baculoviral IAP repeat-containing 5 (*BIRC5*), and the most downregulated TNF receptor superfamily member 11B (*TNFRSF11B*, 16.34-fold). In conclusion, the data obtained indicate that the two cell lines were markedly different in the anticancer effect and mechanisms of oleuropein’s ability to alter apoptosis-related gene expressions. The results obtained from this study should also guide the potential utilization of oleuropein as an adjunct therapy for TNBC to increase chemotherapy effectiveness and prevent cancer progression.

## 1. Introduction

Breast cancer (BC) is the second leading cause of cancer death among women in the United States [1]. Incidence of BC culminated in more than 279,000 new cases in the United States and an estimated over 42,000 death cases [2]. The most aggressive type of BC is triple-negative breast cancers (TNBC), characterized by the lack of expression of estrogen receptors (ER), progesterone receptors (PR), and the lack of the overexpression of the human epidermal growth factor receptor 2 (HER2) in the breast tumor [3]. TNBC is more common in African American (AA) women as compared to Caucasian American (CA) women [3,4,5]. Furthermore, a higher death rate has been found in AA women than in CA women [3,6]. Although many therapeutic strategies have been developed, the aggressive and metastatic features of BC remain unresolved issues. The currently used therapeutic approaches are based on targeting receptor expression, in particular, ER, PR, and human epidermal growth factor receptor2 (HER2) [7]. Unfortunately, this approach is hindered in the TNBC cells [8], allowing chemotherapy to be the leading systemic therapeutic option for this BC subtype [9]. However, various challenges are still obstructing triple-negative treatments [10], with higher metastasis, earlier recurrence, and worse outcomes compared to the non-TNBC.

Among cancer cells’ most characteristic feature is their high proliferation rate, enhanced by rapid cell cycle progression [11] to protect tumors from DNA damage. Indeed, the signaling pathways mediating cell cycle arrest and apoptosis are profoundly diminished in cancer cells [12]. Hence, before DNA repair is complete, revoking cell cycle checkpoints can stimulate the apoptotic cascade, leading to transcription inhibition and cell death [13]. It has become evident that many traditional chemotherapeutic agents’ ultimate function is achieved through such a mechanism regulating cell cycle progression to enhance the programmed cell death.

In the multicellular organism, apoptosis is a crucial molecular process for maintaining cellular homeostasis. Upon apoptosis induction, caspases are expressed to orchestrate the apoptotic most distinct stages of the mechanism—the initiator and executioner phases [14]. The intrinsic (mitochondria-mediated) and extrinsic (death receptor-mediated) apoptosis pathways are the well-known initiation pathways that activate caspases, and both lead to the execution phase of apoptosis. Meanwhile, the extrinsic pathway involves ligands, mainly tumor necrosis factor α (*TNF-α*) and FAS ligand, with their death receptors [15,16]. The mitochondria-mediated intrinsic apoptosis pathway controls proapoptotic and antiapoptotic proteins [17]. Cancer cells resisting apoptosis in different ways, including reducing caspases expression, impair death receptor signaling, or disturb the balance of proapoptotic and antiapoptotic proteins [18].

On the other hand, olive trees (*Olea europaea*; family Oleaceae) had been used for many centuries as a traditional herbal drug in Mediterranean and European countries. [19]. Olive leaves also showed anticancer properties [8,20], in addition to other pharmacological properties, including antioxidant [21], neuroprotective, cardioprotective [22,23], hepatoprotective, antidiabetic, hypoglycemic [24,25,26], anti-inflammatory [27], antiviral, and antibacterial [28,29], in addition to the anti-atherogenic effects [30]. In particular, olive products, particularly extra virgin olive oil, hold promise in various health issues [31]. The relationship between olive oil consumption and the reduced risk of cancer was previously evident in different types of cancer, including breast [32,33], prostate [34], lung [35], liver [36], and colon cancer. The medicinal properties of olive oil are specifically attributed to carotenoids, tocopherols, and the most beneficial phenolic compounds.

The compound oleuropein (OL) is the most prominent polyphenol component of olives and leaves [8]. Previous in vivo and in vitro studies suggested the potential anticancer effect of OL. Certainly, the compound has demonstrated cytotoxic, anti-proliferative, apoptotic, and anti-metastatic effects in various types of cancers, including breast [37,38,39,40], colon [41,42], liver [43], prostate [44,45], pancreatic [46], thyroid [47], osteosarcoma [48], leukemia [49], neuroblastoma [50], mesothelioma [51], and glioblastoma cancer [52]. Furthermore, OL appears to synergize doxorubicin action against breast tumor xenografts [53], indicating that OL was absorbed, metabolized, and infiltrated into different organs of the mouse body [54,55]. 

Manipulation of the tumor microenvironment is a promising target in cancer therapy [56]. Myriads of in-vivo and in-vitro studies have emphasized the interaction of OL with different metabolic pathways that mediate cancer. OL was found to reduce inflammatory angiogenesis by suppressing different MMPs family members’ expression and the vascular endothelial growth factor (VEGF) by reducing the pro-inflammatory enzyme COX2 [57]. These effects are accompanied by the activation of the nuclear factor, NF)-κB, and reduced intracellular reactive oxygen species levels [58]. In BC, OL was also found to prevent cancer metastasis by attenuating MMPs gene expression and upregulating the expression of the tissue inhibitors of metalloproteinases (TIMPS) genes [40]. Moreover, OL modulates the inflammatory pathway and inhibits the Toll-like receptor (TLR) signaling by downregulating NOS, COX2, ERK1/2, JNK, and nuclear factor of kappa light polypeptide gene, as well as the pro-inflammatory cytokine interleukin 6 (IL-6), interleukin 1β (IL-1β), and the gene AP-1 [59].

The previously reported anticancer effects of OL—at different concentrations up to ~4500 µM—on different BC cell models have included MCF-7, T47D, SKBR3, and MDA-MB-231 cell lines [37,38,39,60,61,62,63]. However, the OL effect on MDA-MB-468 cells representing TNBC in AA women has not been addressed. Hence, the current study was designed to compare the possible anticancer mechanisms of the polyphenolic compound OL on MDA-MB-468 and MDA-MB-231 cell lines. In this study, the two different TNBC cell lines were studied for cell growth, proliferation, cell cycle progression, and apoptosis. We hypothesized in this investigation that the response of the two racially different cell lines to OL might be exhibited through different apoptosis-related signaling pathways by impacting the expression of various genes controlling these events.

## 2. Materials and Methods

### 2.1. Reagents and Chemicals

Oleuropein (purity ≥ 98%), Alamar Blue^®^, the resazurin fluorescence dye solution, and cell culture water were purchased from Sigma-Aldrich (St. Louis, MO, USA). An Annexin V-FITC Apoptosis Detection Kit Plus (cat. no. 68FT-Ann VP-S) was obtained from RayBiotech (Norcross, GA, USA). Propidium Iodide Flow Cytometry Kit (cat. no. ab139418) was purchased from Abcam (Cambridge, MA, USA). A DNA-free™ kit (cat. no. AM1907) was purchased from Thermo Fisher Scientific Inc. (Waltham, MA, USA). An iScript™ cDNA Synthesis kit (cat. no. 170-8890), SsoAdvanced™ Universal SYBR^®^ Green Supermix, and the Human apoptosis PCR array (SAB Target List) H96 were purchased from Bio-Rad Laboratories (Hercules, CA, USA).

### 2.2. Cell Culture and Media 

Two TNBC cell models, MDA-MB-231 (ATCC^®^ HTB-26™) and MDA-MB-468 (ATCC^®^ HTB-132™) cells, were purchased from the American Type Culture Collection (ATCC; Manassas, VA, USA). Cell culture media, as well as cell supplements, were purchased from ATCC, VWR International (Radnor, PA, USA), Santa Cruz Biotechnology Inc. (Dallas, TX, USA), and Thermo USA Scientific (Ocala, FL, USA). TNBC adherent cells were grown as monolayers in 75-mL tissue culture flasks in a humified incubator at 37 °C and 5% CO_2_. The complete growth Dulbecco’s Modified Eagle Medium (DMEM), containing 4 mM l-glutamine, was supplemented with 10% heat-inactivated fetal bovine serum (FBS) and 1% penicillin/streptomycin salt solution (100 U/mL and 0.1 mg/mL, respectively). Cells were washed with Dulbecco’s phosphate-buffered saline (DPBS) and subculture as required with trypsin/EDTA (0.25%). DMEM supplemented with 2.5% heat inactivated FBS was used as the experimental media.

### 2.3. Cell Viability Assay 

Alamar Blue^®^ (AB^®^) fluorometric assay was used to measure the cytotoxic effect of OL on two TNBC cell lines; MDA-MB-231 and MDA-MB-468 cells. Briefly, both cell lines were plated at 2 × 10^4^ cells/100-µL/well in 96-well plates and placed overnight in a cell culture incubator of 5% CO_2_ at 37 °C. At the same time, wells containing experimental media without cells were used as blanks. OL was solubilized in cell culture water and aliquoted before freezing at −20 °C. The working solution was always prepared fresh on an experimental day. Next to overnight incubation, another 100 µL of the experimental media, with or without OL, was added to the corresponding wells at concentration ranges from 0 to 800 µM for MDA-MB-231 cells and from 0–400 µM for MDA-MB-468 cells. At the end of the 48 h exposure period, all plates were subjected to AB^®^ assay, as previously mentioned [64], by adding 20 µL of AB^®^ reagent to each well and re-incubating the plates at 37 °C and 5% CO_2_ for 24 h. Finally, the reduced resazurin dye was measured at an excitation/emission wavelength of 530/590 nm using a Synergy HTX Multi-Mode microplate reader (BioTek Instruments Inc., Winooski, VT, USA). Each experiment was repeated three times.

### 2.4. Cell Proliferation Assay

The antiproliferative potency of OL was examined following the above cell viability assay. However, the number of cells under investigation was 1 × 10^4^ cells/100 µL/well. We also treated cells for 72 and 96 h with OL at concentrations ranging from 0 to 350 µM for MDA-MB-231 cells and 0 to 250 µM for MDA-MB-468 cells.

### 2.5. Cell Cycle Analysis 

Flow cytometric analyses were performed to evaluate the effects of OL on cell cycle distribution in two TNBC cell models. Briefly, both MDA-MB-231 and MDA-MB-468 cells were seeded at 1.5 × 10^6^ cells/T25 cell culture flasks and kept overnight. Cells were then treated with OL for 48 h at concentration levels 0–400 µM for MDA-MB-231 and 0–150 µM for MDA-MB-468 in a final volume of 6 mL/flask. Floating and attached cells were harvested, centrifuged, washed in DPBS, and fixed in cold 70% ethanol. The suspended cells were pelleted, washed in DPBS, gently resuspended in 1X propidium iodide (PI) + RNase staining solution, and incubated in the dark for 30 min at 37 °C. Lastly, cells were analyzed using a FACSCalibur flow cytometer (BD Biosciences, San Jose, CA, USA). 

### 2.6. Apoptosis Assay 

The apoptotic effect of OL was established in MDA-MB-231 and MDA-MB-468 cells using the previously described protocol [65]. Briefly, in two separate sets, MDA-MB-231 and MDA-MB-468 cells were plated at 5 × 10^5^ cells/well in 6-well plates and incubated overnight under 37 °C and 5% CO_2_ humidified atmosphere. Cells were treated with OL at optimized concentrations ranging from 0 to 700 µM for MDA-MB-231 cells and 0 to 300 for MDA-MB-468 cells. Respective control cells were exposed to only experimental media. After 48 h, cells from each well were collected by gentle trypsinization, pelleted, and washed twice in DPBS. Cell pellets were then suspended in 500 µL of 1X Annexin V binding buffer, gently vortex, and labeled with 5 µL each of Annexin V-FITC and PI. The apoptotic effect was quantified within 10 min, using a FACSCalibur flow cytometer (BD Biosciences, San Jose, CA, USA). For each sample, the flow cytometer collects 1 × 10^4^ events. Unstained cells are considered alive, while cells stained with Annexin V are undergoing apoptosis. Cells that are stained with both reagents are considered at the late apoptosis or necrosis phase. 

### 2.7. Gene Expression Quantification 

#### 2.7.1. RNA Extraction by TRIzol

For each TNBC cell line, two T-75 flasks with 6 × 10^6^ cells designated for the control and treated cells were placed overnight at 37 °C, and 5% CO_2_ humidified incubator. Based on the cell viability and apoptosis assays data, each cell line was treated with OL at a concentration correlated to the IC_50_ value that shows an insignificant necrotic effect (500 µM in MDA-MB-231 cells and 250 µM in MDA-MB-468 cells) [66,67,68]. Control cells were exposed only to the experimental media. After a 48-h incubation period with/without OL, the cells from each flask were collected, centrifuged for 5 min at 1000 rpm, and the cell pellets were washed twice with DPBS. As recommended by the manufacturer, the total RNA was extracted from each sample by homogenization with 1 mL of TRIzol reagent. The phase separation step was completed by adding 0.2 mL of chloroform to each sample, vortexed for 30 s, and incubated for 3 min at room temperature. The samples were then centrifuged at 10,000× *g* for 15 min at 8 °C. RNA rich upper aqueous phase was aspirated into another centrifuge tubes, combined with 500 µL of isopropyl alcohol to precipitate the RNA. The obtained RNA pellets were then carefully washed with 75% ethanol, air-dried for 10 min, dissolved in nuclease-free water (~30–50 µL), and stored at −80 °C freezer. 

#### 2.7.2. Complementary DNA (cDNA) Synthesis

The purity and the concentration of the dissolved RNA were evaluated for each sample using a NanoDrop spectrophotometer (NanoDrop Technologies; Thermo Fisher Scientific Inc.). Following the manufacturer’s protocol, RNA (5 µg/mL) was incubated with a 1X DNase cocktail for 30 min at 37 °C, and the reaction was stopped by adding DNase inactivator. The samples were again centrifuged at 9000 rpm for 3 min to precipitate unwanted DNA. For first-strand cDNA synthesis, reverse transcription (RT) of the purified RNA samples was performed using the iScript™ cDNA Synthesis kit. Briefly, in each well of the 96-well PCR plates, 5 µL of the DNA-free supernatant was combined with 9 µL of nuclease-free water and 6 µL of advanced reaction mix reverse transcriptase cocktail. Lastly, the PCR plates were subjected to the RT reaction as follows: RT for 20 min at 46 °C, and RT inactivation for 1 min at 95 °C. The obtained cDNA for each sample was kept in a −80 °C freezer for later PCR run.

#### 2.7.3. Quantitative Reverse Transcription-Polymerase Chain Reaction (qRT-PCR) Apoptosis Array 

The 96-well apoptosis array was loaded with 10 µL each of the synthesized cDNA (2.3 ng) and SsoAdvanced™ Universal SYBR^®^ Green Supermix for a final volume of 20 µL/well. The plate was then placed on a low-speed shaker for 5 min and centrifuged at 1000× *g* for 1 min. Bio-Rad CFX96 Real-Time System (Bio-Rad Laboratories, Hercules, CA, USA) was used to establish the fluorescent quantification in PCR. The cDNA was amplified under 39 thermo-cycling of denaturation, starting with 30 s activation at 95 °C, 10 s denaturation at 95 °C, and 20 s annealing at 60 °C. The final extension step was completed at 65 °C for 31 s. For each cell line, qRT-PCR data were validated by three independent experiments. 

### 2.8. Statistical Analysis 

GraphPad Prism 6.2 software (GraphPad Software Inc., San Diego, CA, USA) was used to analyze the data for the current study. All data points present the average of at least two independent experiments and are expressed as the mean ± SEM. For the viability and proliferation assays, the IC_50_ value of each experiment was determined by a nonlinear regression model of log (inhibitor) vs. normalized response-variable slope on the software with the R^2^ best-fit of lowest 95% confidence interval. The average of IC_50_ +/− SEM of the biological replicates was calculated on an Excel sheet. Apoptosis and cell cycle distribution acquisition and data analysis were presented using CellQuest software (BD Biosciences, San Jose, CA, USA). Gene expression quantification was analyzed using the CFX 3.1 Manager software (Bio-Rad Laboratories, Hercules, CA, USA). The significance of the difference was determined using one-way or two-way analysis of variance (ANOVA) followed by Bonferroni’s multiple comparison test. An unpaired Student *t*-test was used for comparing two data sets. Generally, a difference was considered significant at * *p* < 0.05 (as indicated in the figures and legends). 

## 3. Results

### 3.1. Oleuropein Decreases the Cell Viability of Triple-Negative Breast Cancer Cells

**Figure 1:**
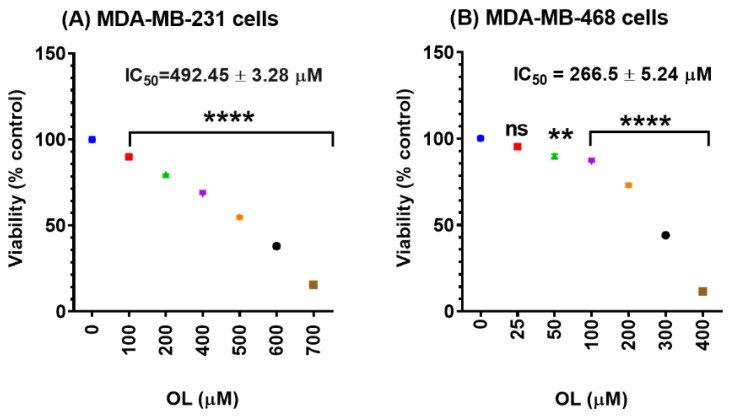
Cytotoxic effect of oleuropein (OL) on (**A**) MDA-MB-231 and (**B**) MDA-MB-468. Triple-negative breast cancer (TNBC) cell lines were seeded at 2 × 10^4^ cells/100-µL/well in 96-well plates and treated for 48 h with increasing concentrations of OL ranges of 0–800 µM in MDA-MB-231 cells and 0–400 µM in MDA-MB-468 cells in a final volume of 200 µL of the experimental media. The graph illustrates the cell viability data expressed as percentages of cell survival compared to the control. The data points represent the average ± standard error of the mean (SEM) of three independent studies, *n* = 5. One-way analysis of variance (ANOVA) followed by Bonferroni’s multiple comparisons test was used to determine the significance of the difference between the control vs. treated groups. The difference was considered significant at ** *p* < 0.01 and **** *p* < 0.0001. ns, nonsignificant.

To investigate the anticipated anticancer effect of OL in TNBC, we evaluated cell viability in two racially different TNBC cell models, MDA-MB-231 and MDA-MB-468 cells, at concentration ranges of 0–700 and 0–400 µM of OL, respectively. The dose–response curve implies a higher sensitivity (~2-fold more) of MDA-MB-468 cells to the compound, compared with its counterpart cell line, MDA-MB-231 (IC_50_ = 492.45 ± 3.28 µM for MDA-MB-231 cells (Figure 1A) and 266.5 ± 5.24 µM for MDA-MB-468 cells (Figure 1B) at a 48 h exposure period. A highly significant cytotoxic effect (*p* < 0.0001) was also identified in both cell lines, at 100–700 µM in MDA-MB-231 and 100–400 µM in MDA-MB-468. A small but significant decrease in cell viability was also detected in MDA-MB-468 cells at 50 µM (*p* ˂ 0.01).

### 3.2. Oleuropein Inhibition of the Growth Rate in Triple-Negative Breast Cancer Cells

**Figure 2:**
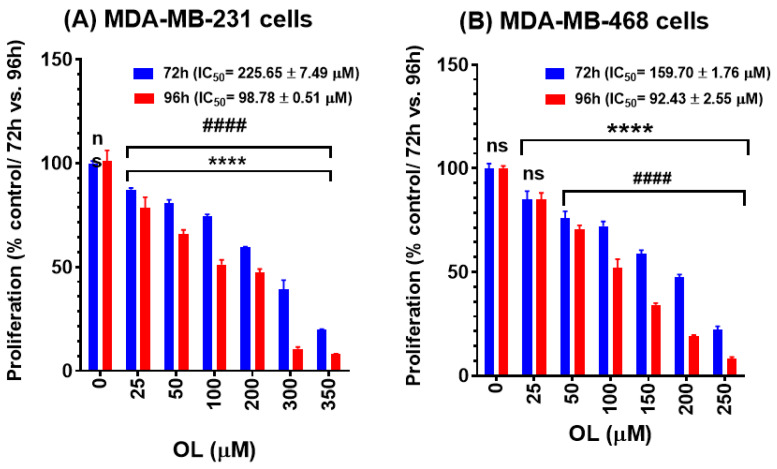
Antiproliferative effect of oleuropein (OL) in (**A**) MDA-MB-231 and (**B**) MDA-MB-468 TNBC cell lines. Both cell lines were seeded in 96-well plates at 1 × 10^4^ cells/100-µL/well and incubated for 72 and 96 h with OL at concentration ranges of 0 to 350 µM or 0–250 µM in MDA-MB-231 and MDA-MB-468 cells, respectively. Each data point presents the average ± SEM of two independent experiments; *n* = 5 each. One-way and Two-way ANOVA tests, followed by Bonferroni’s multiple comparisons test, were used to calculate the p-values for the difference between control vs. 72 or 96 h exposure (*) or between the different exposure periods (#), respectively. ****/#### *p* < 0.0001 indicate a statistically significant difference. ns, nonsignificant.

The antiproliferative effects of OL were assessed using AB^®^ assay to measure the indirect cytotoxic effect of the compound at a longer exposure period as implied by the reduced growth rate in MDA-MB-231 and MDA-MB-468 cells. The antiproliferative effects of the compound were demonstrated in a dose- and time-dependent response compared to the control cells (Figure 2). Overall, the data obtained were consistent with the viability study data. MDA-MB-468 cells were more sensitive to the compound than MDA-MB-231 cells. In both tested cell lines, OL significantly inhibited cell proliferation (*p* < 0.0001) at the 72 and 96 h exposure periods vs. the control (Figure 2A,B). Furthermore, the compound induced antiproliferative effects at 72 vs. 96 h incubation periods. Meanwhile, the highly significant (*p* < 0.0001) inhibition of cell proliferation between these two periods was found at all tested concentrations of OL in both cell types (Figure 2A,B); a nonsignificant effect was detected in MDA-MB-468 cells at the lowest tested concentration (25 µM) of the compound (Figure 2B). Indeed, cell proliferation inhibition is verified by the decrease in the IC_50_ values from 159.70 to 92.43 µM in MDA-MB-468 cells and from 225.65 to 98.78 µM in MDA-MB-231 cells at the 72 vs. 96 h period of exposure. Interestingly, at the 96-h incubation period, the minor difference between the obtained IC_50_ values for both cell lines suggest a similar response to the compound. 

### 3.3. Oleuropein-Induced Cell Cycle Arrest in Triple-Negative Breast Cancer Cells

**Figure 3:**
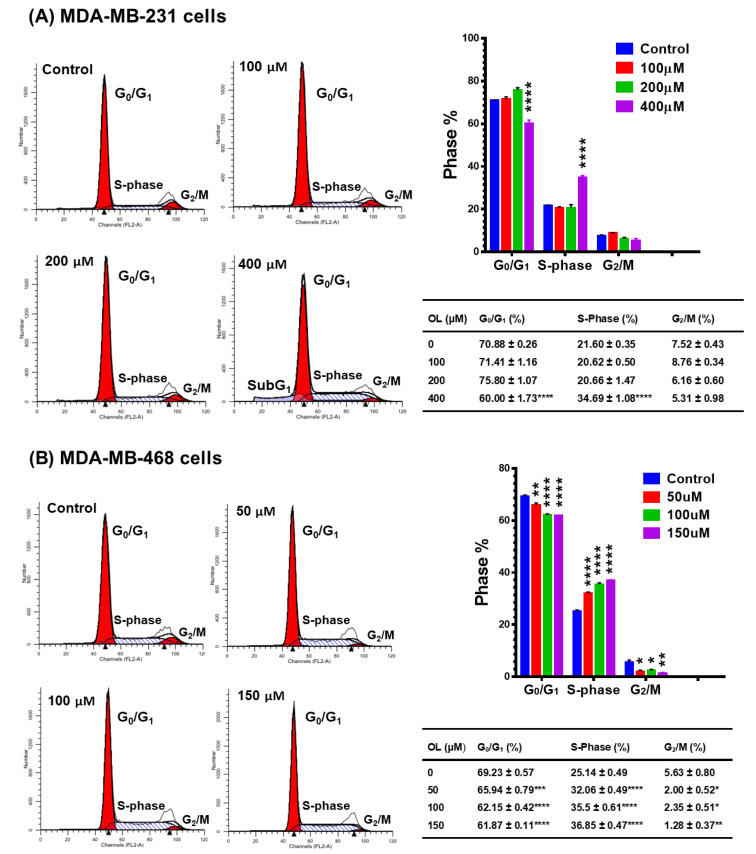
Effect of oleuropein (OL) on cell cycle distribution in (**A**) MDA-MB-231 and (**B**) MDA-MB-468 TNBC cell lines. Both cell lines were treated for 48 h with OL at three different doses between 0 and 400 µM in MDA-MB-231 cells and 0 and 150 µM in MDA-MB-468 cells. PI fluorescence histograms show flow cytometry analysis of cell distribution. FACSCalibur software was used to analyze the percentage of different phases in OL-treated samples vs. control. One-way ANOVA, followed by Bonferroni’s multiple comparisons test, was used to determine the *p*-values for the difference between the control and treated cells at different cell cycle phases. The difference was considered significant at * *p* < 0.05, ** *p* < 0.01, *** *p* < 0.001, and **** *p* < 0.0001.

To comprehend the mechanism provoking the cytotoxic and antiproliferative effects of OL, flow cytometric analysis using PI staining was performed to evaluate the early effect of the compound on cell cycle distribution after 48 h exposure at 0–400 µM in MDA-MB-231 (Figure 3A) and 0–150 µM in MDA-MB-468 (Figure 3B). The data show that DNA fluorescent content frequency histograms indicate the ability of OL to impact cell cycle progression. However, the two cell models showed a similar response; the susceptibility of MDA-MB-468 to the compound was ~3-folds higher than that for MDA-MB-231 cells. Notably, the three cell cycle phases were significantly affected (*p* < 0.05–*p* < 0.0001) in MDA-MB-468 cells at all tested OL concentrations. Meanwhile, MDA-MB-231 showed a significant response only at the highest tested concentration of the compound (400 µM). The data showed that the significant increase in S-phase (*p* < 0.0001) was accompanied by a decrease in G_0_/G_1_ (*p* < 0.001–*p* < 0.0001). Indeed, a more than 10% increase in S-phase was measured in both OL-treated cell types, compared to the control cells (34.69 ± 1.08 vs. 21.60 ± 0.35 in MDA-MB-231 and 36.85 ± 0.47 vs. 25.14 ± 0.49 in MDA-MB-468). These events were induced in MDA-MB-231 cells at 400 µM, accompanied by an increase in the number of dead cells (Sub G1, ~15%), as observed to the left of the G0/G1 peak in Figure 3A. Meanwhile, the same S-phase arrest was measured in MDA-MB-468 cells at 150 µM and resulted in a significant reduction in the G2/M phase (*p* < 0.01). Together with proliferation data, these results point to a different mechanism of OL in MDA-MB-468 that is more effective than the mechanism by which OL impacts the MDA-MB-231 cell line.

### 3.4. Oleuropein-Stimulated Apoptotic Effect in Triple-Negative Breast Cancer Cells

**Figure 4:**
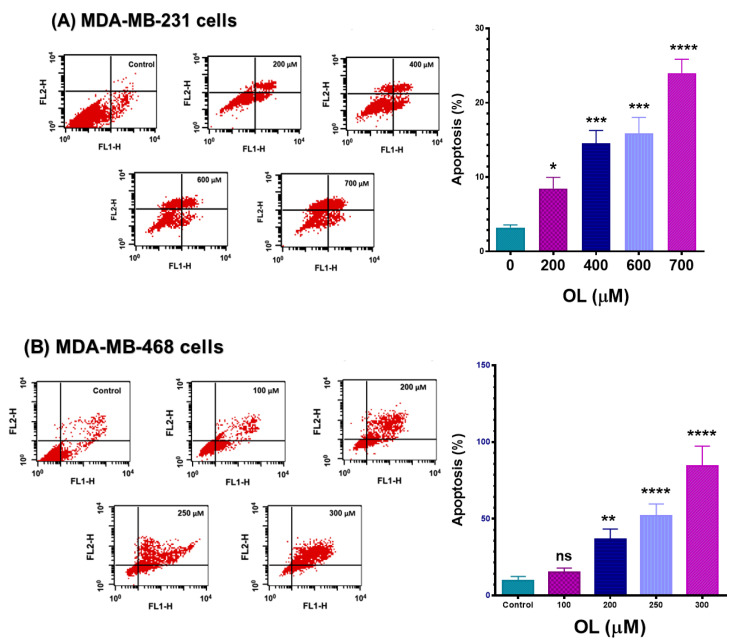
Apoptotic effects of oleuropein (OL) in (**A**) MDA-MB-231 and (**B**) MDA-MB-468 TNBC cell lines. TNBC cells were exposed to OL for 48 h at concentrations ranging from 0 to 700 µM in MDA-MB-231 cells and 0–300 µM in MDA-MB-468 cells, while untreated control cells were incubated only with experimental media. Treated and control cells were labeled using an Annexin-V-FITC apoptosis kit. The percentage of apoptotic and necrotic cells in each sample was analyzed using CellQuest software. Each data point in the bar graphs represents the mean ± combined percentage of early and late apoptotic cells and the SEM of two independent studies with *n* = 3 each. The significance of the difference between each treatment vs. control was calculated using one-way ANOVA, followed by Bonferroni’s multiple comparisons test. * *p* < 0.05, ** *p* < 0.01, *** *p* < 0.001, and **** *p* < 0.0001 indicated the statistically significant difference. ns, nonsignificant. FL1-H, Annexin V-FITC, and FL2-H, propidium iodide

Further flow cytometry study was conducted, using an Annexin V-FITC apoptosis kit to decipher a possible mechanism underlying the varied antiproliferative, cell cycle arrests, and possible apoptotic effect in OL-treated TNBC cells. The apoptotic effect of OL at the 48-h exposure period in both MDA-MB-231 and MDA-MB-468 cell lines was assessed, as illustrated in Figure 4. The flowcytometric analysis suggested that apoptosis could be among the events leading to cell death. Indeed, the data showed a significant dose-dependent increase (*p* < 0.05–*p* < 0.0001) in the percentage of apoptotic cells compared with the untreated control (Figure 4A,B). The apoptotic effect (early and late apoptosis) of the compound was more profound in MDA-MB-468 cells (~4-fold-more) than its counterpart; MDA-MB-231 cells showed a slower increase in apoptosis. When exposed to 300 µM of OL, a profound 80% of the MDA-MB-468 cells were in the apoptotic phase; meanwhile, 25% of MDA-MB-231 cells analyzed exhibited apoptotic effects at 700 µM. While necrotic cells appeared in MDA-MB-231 cells, a negligible percentage of necrosis was detected in its counterpart, MDA-MB-468 cells. Thus, the obtained data suggested apoptosis as the anticipated primary mode of cell death in OL-treated MDA-MB-468 cells of the AA origin. On the other hand, MDA-MB-231 exhibited higher resistance to apoptosis and tended to undergo necrosis.

### 3.5. Gene Expression Alteration in OL-Treated Triple-Negative Breast Cancer Cells

**Figure 5:**
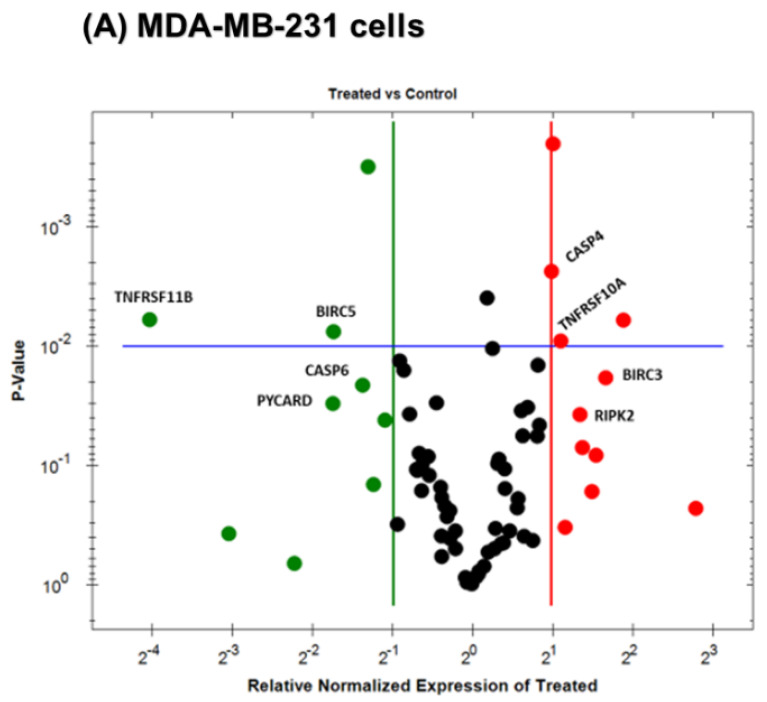

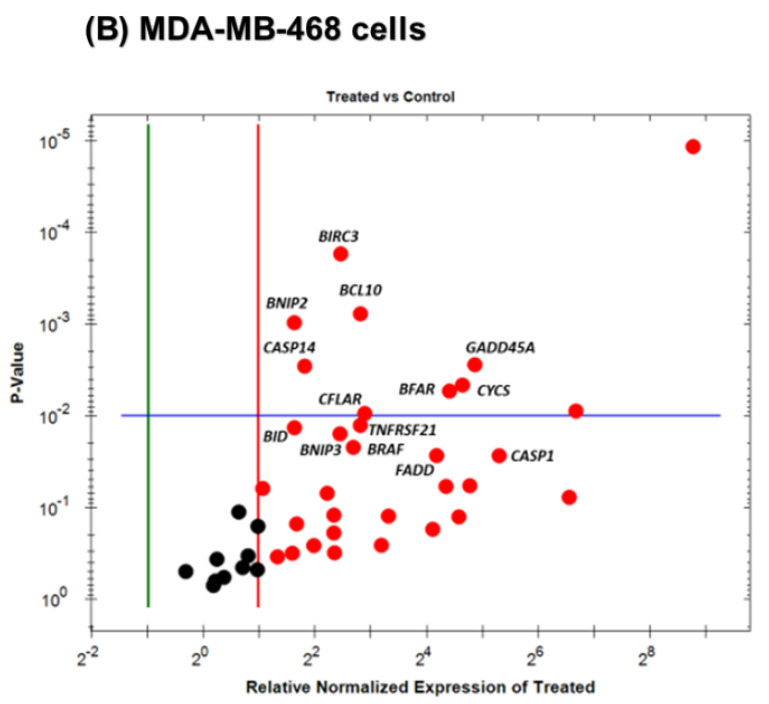
Classification of apoptosis-related gene expression induced by oleuropein (OL) in (**A**) MDA-MB-231 and (**B**) MDA-MB-468 TNBC cells. A volcano plot was performed to categorize and display upregulated (red), repressed (green), or unchanged (black) mRNA gene expression after a 48 h treatment period with 500 µM in MDA-MB-231 cells and 250 µM in MDA-MB-468 cells. The upregulated apoptosis-related genes were highly recognizable in MDA-MB-468 cells more than in MDA-MB-231 cells; meanwhile, green spots were only evident in MDA-MB-231 cells.

In further analysis, quantitative real-time PCR was used to assess the transcriptomic level of apoptosis-related genes in OL-treated TNBC cells. Each cell line was treated with a distinct concentration related to the IC_50_ value (500 µM in MDA-MB-231 cells and 250 µM in MDA-MB-468 cells, Figure 1). These chosen doses did not cause a considerable necrotic effect in the flow cytometry apoptosis analysis (Figure 4). Profiling the normalized mRNA expression for cells under investigation provided intuition into OL’s impact on various apoptosis-linked genes; however, we presented only the significantly altered mRNAs. Taken together, the red dots represent the upregulated genes (Figure 5A,B); meanwhile, the green dots refer to the downregulated genes that were observed only in MDA-MB-231 cells (Figure 5A). For both cell lines, the black dots indicate the unchanged gene expression (Figure 5A,B).

Remarkably, MDA-MB-468 cells showed superiority over MDA-MB-231 cells regarding the number of altered genes and the fold-change (Table 1). Hence, the results may explain the higher anticancer potency of OL against MDA-MB-468 cells. A total of 14 genes expressed a significant increase (*p* < 0.05–*p* < 0.001) in their mRNA level (between 3.07 and 39.81-fold) upon MDA-MB-468 exposure to 250 µM of OL. Among these upregulated genes, caspase 1 (*CASP1*) had the highest fold increase (39.81-fold), followed by four genes with upregulations ranged from 29.09-18.31-fold, including growth arrest and DNA damage-inducible 45 alpha (*GADD45A*), cytochrome c somatic (*CYCS*), bifunctional apoptosis regulator (*BFAR*), and Fas-associated via death domain (*FADD*). Additionally, six genes were significantly impacted with 5.36–7.43-fold upregulation: CASP8 and FADD-like apoptosis regulator (*CFLAR*), tumor necrosis factor (TNF) receptor superfamily 21 (*TNFRSF21*), B-cell lymphoma/leukemia 10 (*BCL10*), B-Raf proto-oncogene (*BRAF*), a baculoviral inhibitor of apoptosis (IAP) repeat-containing 3 (*BIRC3*), and BCL-2 interacting protein 3 (*BNIP3*). The least upregulated three genes with a 3–4-fold increase were caspase 14 (*CASP14*), BCL-2 interacting protein 2 (*BNIP2*), and BH3 interacting domain death agonist (*BID*).

At 500 µM concentration level, oleuropein impacted the regulation of 8 genes in MDA-MB-231 cells (Table 1). However, the fold-change was smaller compared to its counterpart, MDA-MB-468 cells. The mRNA levels were significantly upregulated in four genes (*p* < 0.05–*p* < 0.01) with a 1.98–3.18-fold increase, including (*BIRC3*), receptor-interacting serine/threonine kinase 2 (*RIPK2*), TNF receptor superfamily 10A (*TNFRSF10A*), and caspase 4 (*CASP4*). On the other hand, another four genes were downregulated with TNF receptor superfamily member 11B (*TNFRSF11B*) being the most profoundly downregulated (−16.34-fold), followed by three slightly downregulated genes, including baculoviral IAP repeat-containing 5 (*BIRC5*), pyrin domain (PYD), and caspase recruitment domain (CARD)-containing (*PAYCARD*), and caspase 6 (*CASP6*) was the least repressed (2.60-fold decrease). 

*BIRC3* mRNA was the only significantly upregulated gene in both cell types; yet the fold increase in MDA-MB-468 cells was higher than that in MDA-MB-231 cells (5.50 vs. 3.18-fold). Our data constructively draw attention to the different response mechanisms exhibited by two racially different TNBC cells in response to OL.

## 4. Discussion

The relationship between olive oil consumption and the reduced risk of breast cancer has been previously established [31,32]. Olive oil medicinal properties were attributed to its natural polyphenolic contents that have received considerable attention for their use as a cancer chemopreventive and chemotherapeutic agents [31,32]. The compound oleuropein (OL) is the most prominent polyphenol component of olives and leaves [8]. Meanwhile, the cell cycle-mediated apoptosis pathway is considered a rational approach for enhancing tumor sensitivity in coordination with anticancer agents [69]. The current study provides an analysis of apoptotic proteins participating in the anticancer effect OL. The results suggest two markedly different mechanisms in altering apoptosis-related gene expressions between the two genetically different TNBC cell lines: MDA-MB-231 and MDA-MB-468. These differences suggest that the specific therapeutic approach for TNBC must take into account the variability in the genetic background. 

The data obtained in our study indicate the anticancer effects of OL in the two different triple-negative breast cancer (TNBC) cell lines. The polyphenol OL was found to induce cytotoxic effects and inhibit the proliferation rate in both MDA-MB-231 and MDA-MB-468 cell lines. The cytotoxicity studies (Figure 1), proliferation assays (Figure 2), and cell cycle distribution analysis (Figure 3) indicated the different responses in these two cell lines. The results obtained show that MDA-MB-468 cells were more sensitive to OL than the MDA-MB-231 cell line. Compared to the currently used chemotherapy drugs, our recently published study [64] indicated the IC_50_ values for TNBC cells following 72 h of exposure to doxorubicin as 1.69 ± 0.11 and 0.23 ± 0.003 µM in MDA-MB-231 and MDA-MB-468 cells, respectively. Even though the compound induced S-phase arrest in both cell lines, MDA-MB-468 cells were more susceptible to apoptosis compared to MDA-MB-231 cells (Figure 4). Our gene analysis investigation demonstrated OL’s ability to impact a different subset of genes mediating apoptosis in these TNBC cell lines (Figure 5).

Furthermore, the apoptosis-related genetic quantification indicates the higher vulnerability of MDA-MB-468 to OL than the MDA-MB-231 cell line, as indicated by a significantly higher fold-increase in the number of altered genes (Table 1). The ability of OL to suppress cell proliferation and stimulate apoptosis through S-phase cell cycle arrest in both cells is consistent with previous studies on BC cells at 200 µM [70]. Our data are also supported by a previous study that linked S-phase cell cycle arrest to particular apoptosis-related genes [71].

In the current study, the alteration of gene expression signature suggests the potency of OL to induce apoptosis through intrinsic and extrinsic pathways. Gene expression profiling in our study demonstrated that OL induced higher apoptosis-related transcriptional impacts in MDA-MB-468 cells than in MDA-MB-231 cells. Indeed, the most 14 significantly altered genes that were detected in MDA-MB-468 cells were all upregulated. In comparison, in MDA-MB-231, genes were altered with twice the concentration of OL used for MDA-MB-468. Our results suggest that OL caused differential gene expression changes to the TNBC gene, depending on the cell genotype.

Caspases, the cysteinyl aspartate proteases, naturally sustain cellular homeostasis by regulating apoptosis [72,73]. The data analysis from the current study revealed the impact of OL on different caspases. In MDA-MB-468 cells, OL predominantly upregulated *CASP1*, followed by *CASP14*. Instead, *CASP4* was upregulated, while *CASP6* was downregulated in MDA-MB-231 cells (Table 1). The three caspases, *CASP*1, *CASP4*, and *CASP14*, are apoptosis initiator caspases [74,75] that mediate the innate immune responses to cellular stress [76] and stimulate pyroptosis, a type of cell death [77,78]. On the other hand, *CASP14* is involved in cornification, a unique type of programmed cell death other than apoptosis [79,80]. We propose that the anticancer effect of OL on the MDA-MB-468 cell does not rely solely on apoptosis, but also pyroptosis and/or cornification mechanism could also be involved.

*CASP1* is the most upregulated gene (39.81-fold) in OL-treated MDA-MB-468 cells. Unlike healthy breast cells, low expression of *CASP1* in various types of cancer, including BC, is closely associated with decreased apoptosis and cancer cell enhancement [81]. On the contrary, upregulated *CASP1* in fibroblasts induces apoptosis that precedes cell death [82]. Previous reports also suggested that *CASP1* activates *BID* and is involved in the intrinsic mitochondrial apoptotic pathway [83,84]. Hence, our results suggest the substantial contribution of *CASP1* to the observed apoptotic effect in OL-treated MDA-MB-468 TNBC cells and recommend *CASP1* as a novel target in BC patients.

**Figure 6:**
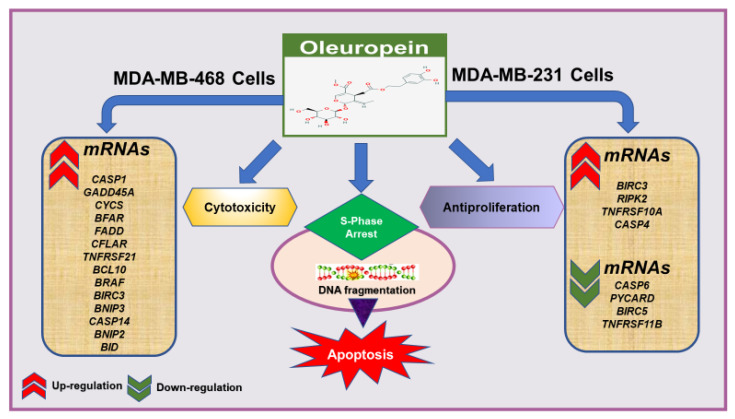
The effect of OL in MDA-MB-231 and MDA-MB-468 TNBC cells.

In MDA-MB-231 cells, OL induced differential responses in *CASP4* and *CASP6* (Figure 6 and Table 1). Our finding is consistent with those previously reported the association between *CASP4* and the endoplasmic reticulum (ER) stress-induced intrinsic apoptotic pathway [85,86,87]. Surprisingly, the downstream effector and executioner, *CASP6*, was downregulated in MDA-MB-231 cells and could be another reason for the weak apoptotic effect in OL-treated MDA-MB-231.

Four members of the TNF receptor superfamily (also known as death receptor, DR) were differently altered in OL-treated TNBC cells. Inversely altered *TNFRSF10A* and *TNFRSF11B* in MDA-MB-231 cells versus the highly upregulated mRNA in *TNFRSF21* and *FADD* in MDA-MB-468 cells. In our study, *TNFRSF11B* was significantly downregulated in OL-treated MDA-MB-231 cells. This gene is expressed in various cancer types and is considered a prognostic marker in MDA-MB-231 TNBC cells [88]. *TNFRSF11B* overexpression has multiple implications in cancer invasion, metastasis, as well as poor prognosis [89,90,91]. This gene also enhances cancer cell survival by resisting apoptosis induced by a member of the TNF superfamily known as TNF-related apoptosis-inducing ligand (TRAIL) [90]. Therefore, the dramatic repression of *TNFRSF11B* expression is suggested to be the main contributor to the apoptotic effect in the OL-treated MDA-MB-231 cell line. 

In MDA-MB-468 cells, the significantly upregulated *FADD* receptor may be involved in the extrinsic and TRAIL-induced apoptotic pathway through caspase activation [92,93] and the vital role in *BID* cleavage [73]. Consistent with previous findings in BC [94], the upregulated expression of *FADD* in MDA-MB-468 could mediate the observed S-phase cell cycle arrest and apoptosis. The upregulated *TNFRSF21* (DR6), which appeared in the treated MDA-MB-468 cells under investigation, was previously reported to induce apoptosis [95,96], possibly through the mitochondria-mediating pathway and Bax interaction [97]. However, a slight but significant augmentation of *TNFRSF10A* (DR4) was measured in MDA-MB-231cells. Consistent with the previous findings, the upregulated *TNFRSF10A* could mediate cell cycle arrest, antiproliferative, and apoptotic effects [98,99].

Three members of the CARD family were significantly altered in OL-treated TNBC cells, including *PYCARD*, *BCL10*, and *RIPK2*. In MDA-MB-231 cells, a downregulation in *PYCARD* mRNA expression was detected. Low expression of *PYCARD* is detected in various types of cancers, including BC, and associated with tumorigenesis [100,101]. On the contrary, the ectopic expression of the gene promotes apoptosis, accompanied by DNA fragmentation and growth inhibition [102]. According to the previous study, *PYCARD* acts as an activator of *CASP1* [103], the upstream positive regulator of CASP6-mediated cell death [104]. The fact that implies the involvement of *PYCARD* attenuation in *CASP6* inhibition. This study also shows that OL-treatment of MDA-MB-231 led to PYCARD downregulation and the consequential unchanged *CASP1* expression. Another CARD-contained gene, *RIPK2*, was upregulated only in MDA-MB-231 cells. In TNBC cells, particularly MDA-MB-231 cells, overexpression of *RIPK2* is a characteristic prognostic biomarker, and it is positively correlated with migration and metastasis and contributes to multidrug resistance [105]. Additionally, overexpression of *RIPK2* activates various cell signaling pathways, including NF-ƙB, JNK, and MAPKs [105,106,107]. 

In MDA-MB-468 cells, *BCL10* mRNA was the only altered member of the CARD family. The proapoptotic gene, *BCL10* [108], triggers apoptosis by activating the apoptotic protease activating factor (Apaf-1)-caspase-9 pathway via a *CASP8*-independent mechanism [109]. Moreover, as an immune signaling adaptor, *BCL10* mediates immune response and could trigger cell cycle arrest [110]. In *BCL10*-transfected BC cells, the overexpression of the gene was linked to apoptosis [109,111]. Collectively, our results are consistent with those previously reported. 

Bcl-2 family members control mitochondrial membrane permeability and are crucial regulators of cell death and cell survival [112]. Four proapoptotic genes belonging to this family were upregulated in OL-treated MDA-MB-468 cells: *BNIP2*, *BNIP3*, and *BID*, together with the dramatically upregulated *CYCY*, which possibly has a dual role (Table 1). The gene *CYCS* can suppress the antiapoptotic proteins and activate the proapoptotic proteins of the BCL2 family, leading to an increase in the mitochondrial membrane permeability and allow more *CYCS* release into the cytosol [113]. 

Structurally, the death-inducing mitochondrial BNIP proteins *BNIP2* and *BNIP3* are characterized by the Bcl-2 homology domain 3 (BH3). The most familiar member, *BNIP3,* also contains a C-terminal transmembrane hydrophobic domain, and both domains are vital in stimulating the mitochondrial intrinsic apoptosis pathway [114,115,116]. Given the previous literature, overexpressed *BNIP3* induces both caspase-dependent [117] and caspase-independent apoptosis, and autophagy [118], in addition to caspases-independent necrosis-like cell death [119]. Furthermore, a relationship between the elevation of *BNIP3* expression, cell cycle arrest, and apoptosis was previously reported [120]. Similarly, the *BID* mRNA upregulation was measured in MDA-MB-468 cells, compared with the respective untreated controls (Table 1). The proapoptotic protein *BID* triggers the intrinsic apoptosis pathway through the permeabilizing mitochondrial membrane to facilitate apoptotic protein release such as *CYCS* and activate caspases [121]. Further reports suggested the role of *BID* in DNA damage-stimulated apoptosis and S-phase cell cycle arrest [71,122] 

The proapoptotic gene, *GADD45A*, was the second most upregulated gene in OL-treated MDA-MB-468 cells, after *CASP1*, which is a p53 and DNA damage response gene. As an inducible stress gene, *GADD45A* regulates many biological processes, including DNA repair, apoptosis induction, and cell cycle regulation [123,124]. Particularly in the aggressive TNBC cells, low expression of *GADD45A* was strongly associated with lacking the three hormonal receptors, ER, PR, and Her2 [125]. Our results are consistent with previous reports from others, where *GADD45A* has been shown to induce antiproliferative effects and S-phase cell cycle arrest [126] and activating both JNK and/or p38 MAPK signaling pathways [124,127]

On the other hand, a multidomain antiapoptotic gene, *BFAR*, was significantly upregulated in MDA-MB-468 cells (~21-fold). *BFAR* was found to inhibit both extrinsic and intrinsic apoptosis pathways to enhance cell growth [128]. In BC, an upregulation of *BFAR* was observed after chemotherapy [129]. These findings may explain the increase in *BFAR* mRNA, following MDA-MB-468 cells exposure to OL, as a resistance mechanism. 

Another upregulated antiapoptotic gene in OL-treated MDA-MB-468 cells is the *BRAF* gene that encodes serine/threonine-protein kinase B-RAF [130]. The mutated *BRAF* gene is a downstream effector in the MAPK signaling pathway, which controls many cellular processes, including cell growth, proliferation, differentiation, apoptosis, and migration [131]. The previous reports may be controversial about the *BRAF* effect in BC [132,133,134], even though more recent study presents the *BRAF* gene as an antiapoptotic agent, which could be exhibited in OL-treated MDA-MB-468 cells to resist apoptosis. However, to comprehend the anticipated mechanism of the *BRAF* gene in apoptosis, further molecular investigations are required.

In OL-treated MDA-MB-468 cells, a significant and high level of the antiapoptotic gene *CFLAR* was expressed. The gene *CFLAR* has many isoforms with controversial roles, either as an apoptosis inducer [135,136] or inhibitors of the death-receptor-induced apoptosis pathway that prevented *CASP8* activation [68,137,138]. Therefore, the increase in *CFLAR* mRNA in our study without any significant alteration of *CASP8* may support the later findings that suggest the antiapoptotic action of the gene. Moreover, OL altered the expression of the antiapoptotic survivin *BIRC3* (cIAP2) and *BIRC5* (survivin). These two members of the inhibitor of apoptosis family (IAP) [139]—with their baculoviral IAP repeat (BIR) domains—play a crucial role in controlling proliferation, differentiation, as well as apoptosis [140,141]. In TNBC—MDA-MB-231 and MDA-MB-468—elevated *BIRC3* and *BIRC5* were found, compared with the normal breast cells [142,143]. These proteins block the apoptosis pathway by inhibiting caspases activation [72,144], leading to aggressive tumorigenesis, metastasis, and a poor clinical outcome [145,146,147]. In our study, the *BIRC3* mRNA expression was significantly upregulated in both cell types under investigation. Interestingly, the *BIRC3* upregulation in MDA-MB-231 was concomitant with repression in *BIRC5*, which leads to cell cycle arrest [148]. According to these previous reports, we suggest *BIRC5* repression in MDA-MB-231 cells as an exhibition of submissive apoptosis.

## 5. Conclusions 

The current study demonstrated the molecular mechanism underlying the apoptotic effect of the polyphenolic compound oleuropein (OL) in TNBC cells, MDA-MB-231, and MDA-MB-468 cells (Figure 6). The compound induced cytotoxic and antiproliferative effects accompanied by apoptosis in response to S-phase cell cycle arrest. Meaningfully, the variation in the molecular profiles between AA and CA BC patients—including protein and gene expressions, somatic mutations, somatic DNA copy number alteration (CNA), and DNA methylation patterns [149], might explain the obtained different genetic response to OL in the two cell lines under investigation. Nonetheless, MDA-MB-468 cells were remarkably more susceptible to the compound than MDA-MB-231 cells were. In MDA-MB-468 cells, many apoptosis-involved genes with a dramatic higher fold increase were measured, including two members of the caspase family (*CASP1* and *CASP14*), proapoptotic genes (*GADD45A*, *BNIP2*, *BNIP3*, *BID*, and *BCL10*), two members of the TNF receptor superfamily (*FADD* and *TNFRSF21*), in addition to *CYCS* and *CFLAR* genes. These genes were previously found to augment apoptosis by triggering intrinsic, extrinsic, or both apoptotic pathways (Figure 6).

For MDA-MB-231 cells, the upregulation of *BIRC3*, *RIPK2*, and the downregulation of *CASP6* and *PYCARD* interpret the weaker apoptotic effect in OL-treated cells. However, apoptosis was mainly endorsed by the downregulation of the antiapoptotic gene *TNFRSF11B* and the survivin *BIRC5* and the minor upregulation of *CASP4*, even with alteration in genes resisting apoptosis induction (Figure 6). OL was found to overcome their effects and induced apoptosis in both TNBC cell models. 

As an in vitro investigation, our study has some limitations. While we examined the anticancer effect of OL in two different BC cells, this study did not investigate normal breast cells or include in vivo studies. The study also lacks measuring the impact of OL on protein levels to support our gene microarray assays. Furthermore, the TNBC cell lines studied, MDA-MB-468 and MDA-MB-231, as basal-like 1 and mesenchymal, respectively—are structurally different. Despite these limitations, the study provided an overview of the molecular mechanisms of oleuropein in the two TNBC genetically diverse cell lines. The results indicated that the two cell lines were markedly different in the anticancer effect and mechanisms of the ability of oleuropein to alter apoptosis-related gene expressions. The results obtained from this study should also guide the potential utilization of oleuropein as an adjunct therapy for TNBC to increase chemotherapy effectiveness and prevent cancer progression.

## Figures and Tables

**Table 1:** A comparative illustration of oleuropein (OL) impacts mRNA gene expression in MDA-MB-231 and MDA-MB-468 TNBC cells after 48 h exposure period.

Control vs. Treated MDA-MB-231 Cells			Control vs. Treated MDA-MB-468 Cells		
Target Gene	Fold (+/−)	*p*-Value	Target Gene	Fold (+)	*p*-Value
*BIRC3*	+3.18	0.0184	*CASP1*	+39.81	0.0272
*RIPK2*	+2.54	0.0374	*GADD45A*	+29.09	0.0028
*TNFRSF10A*	+2.13	0.0090	*CYCS*	+24.87	0.0046
*CASP4*	+1.98	0.0024	*BFAR*	+21.19	0.0054
*CASP6*	−2.60	0.0202	*FADD*	+18.31	0.0271
*PYCARD*	−3.14	0.0302	*CFLAR*	+7.43	0.0094
*BIRC5*	−3.33	0.0076	*TNFRSF21*	+7.06	0.0127
*TNFRSF11B*	−16.34	0.0060	*BCL10*	+6.95	0.0008
			*BRAF*	+6.16	0.0221
			*BIRC3*	+5.50	0.0002
			*BNIP3*	+5.36	0.0157
			*CASP14*	+3.51	0.0029
			*BNIP2*	+3.09	0.0010
			*BID*	+3.07	0.0135

In MDA-MB-231 cells (left panel): a baculoviral inhibitor of apoptosis (IAP) repeat-containing 3 (*BIRC3*), receptor-interacting serine/threonine kinase 2 (*RIPK2*), TNF receptor superfamily 10A (*TNFRSF10A*), and caspase 4 (*CASP4*) were upregulated, while caspase 6 (*CASP6*), pyrin domain (PYD) and caspase recruitment domain (CARD)-containing (*PAYCARD*), baculoviral IAP repeat-containing 5 (*BIRC5*), and TNF receptor superfamily member 11B (*TNFRSF11B*) were repressed. Comparably, in MDA-MB-468 cells (right panel), all genes were upregulated, including caspase 1 (*CASP1*), growth arrest and DNA damage-inducible 45 alpha (*GADD45A*), cytochrome c somatic (*CYCS*), bifunctional apoptosis regulator (*BFAR*), Fas-associated via death domain (*FADD*), CASP8 and FADD-like apoptosis regulator (*CFLAR*), TNF receptor superfamily 21 (*TNFRSF21*), B-cell lymphoma/leukemia 10 (*BCL10*), B-Raf proto-oncogene (*BRAF*), a baculoviral inhibitor of apoptosis (IAP) repeat-containing 3 (*BIRC3*), BCL-2 interacting protein 3 (*BNIP3*), caspase 14 (*CASP14*), BCL-2 interacting protein 2 (*BNIP2*), and BH3 interacting domain death agonist (*BID*), whereas no genes were inhibited.
